# Apical Periodontitis and Blood–Brain Barrier Compromise: Biological Mechanisms and Current Evidence—A Narrative Review

**DOI:** 10.3390/ijms27219594

**Published:** 2026-10-27

**Authors:** Cristina Bucchi, Marcela Hernández, Alejandra Chaparro, Josep Maria de Anta, María José Bordagaray

**Affiliations:** 1 Department of Comprehensive Adult Dentistry, Oral Biology Research Center, Faculty of Dentistry, Universidad de La Frontera, Temuco 4811230, Chile; 2 Department of Oral Pathology and Medicine, Faculty of Dentistry, Universidad de Chile, Santiago 8380492, Chile; 3 Department of Periodontics, Faculty of Dentistry, Universidad de los Andes, Santiago 7550000, Chile; 4 Department of Pathology and Experimental Therapy, Facultat de Medicina, Universitat de Barcelona, 08907 L’Hospitalet de Llobregat, Spain; 5 Department of Conservative Dentistry, Faculty of Dentistry, Universidad de Chile, Santiago 8380492, Chile

**Keywords:** chronic inflammation, bacterial translocation, neuroinflammation, oral–systemic link, endodontic infection

## Abstract

This narrative review explores the potential link and the associated biological mechanisms between apical periodontitis (AP) and blood–brain barrier (BBB) integrity. An electronic literature search to identify publications related to AP and BBB integrity was conducted using the Scopus and PubMed databases. Both experimental and clinical studies were included and qualitatively analyzed. Available evidence shows that serum inflammatory marker levels are elevated in patients with AP compared to healthy controls, including proteins associated with endothelial barrier disruption such as ZO-1 and Claudin-5. Indirect evidence shows that disruption of the BBB can occur due to circulating inflammatory cytokines and pathogen insults. BBB cells express receptors that respond to circulating insults by upregulating pro-inflammatory cytokines, necroptosis, and proteolytic activity, which compromise BBB integrity. Elevated baseline levels of inflammatory markers from other chronic oral diseases, particularly periodontitis, have been linked to an increased risk of neurological disorders. Although similar pathophysiological mechanisms may be involved and recent in vitro experimental evidence suggests that the systemic inflammatory milieu associated with AP may compromise BBB function, direct in vivo and clinical evidence establishing a causal link remains lacking. Although biological plausibility supports a role for AP in BBB disruption, and recent in vitro experimental evidence has provided the first preliminary support for this association, in vivo preclinical studies and prospective clinical longitudinal studies are still needed to establish a clear causal relationship. Given the global burden of neurological disorders, elucidating the potential impact of AP on BBB integrity represents an important avenue for research.

## 1. Introduction

Apical periodontitis (AP) is a highly prevalent inflammatory condition [1] characterized by the destruction of periapical tissues due to infection of the dental pulp and root canal system [2]. It represents a localized immune response to microbial invasion, leading to the formation of periapical lesions aimed at containing the infection. Although traditionally viewed as a disease confined to the periapical region, growing evidence suggests that AP may exert systemic effects through the dissemination of bacteria, Pathogen-Associated Molecular Patterns (PAMPs), and inflammatory mediators into the bloodstream [3,4,5], which may ultimately compromise distant organ barriers, including the blood–brain barrier.

The concept of oral–systemic connections, through systemic dissemination of bacteria and inflammatory mediators [6,7,8], has been extensively explored in relation to periodontal disease, with chronic periodontitis being linked to cardiovascular, metabolic, and neurodegenerative diseases [9,10,11,12]. While the evidence supporting AP systemic implications is not new [13,14], it has received comparatively less attention, despite shared features with periodontitis such as chronic inflammation, polymicrobial dysbiosis, and systemic dissemination of bacterial products [3,4,5]. In addition, the high vascularization of periapical tissues [15] facilitates a close anatomical connection between periapical lesions and the bloodstream, enabling bacteria, their byproducts, and inflammatory mediators to reach the systemic circulation and, consequently, distant organs [3,4,16]. Emerging studies report elevated serum levels of pro-inflammatory cytokines in individuals with AP [3,4,5], supporting the notion that even localized endodontic infections may contribute to systemic inflammatory burden.

One possible systemic target of both of these mechanisms (microbial dissemination and systemic inflammation) is the blood–brain barrier (BBB), a specialized, selectively permeable structure that maintains central nervous system (CNS) homeostasis by regulating the passage of molecules, toxins, and cells between the bloodstream and brain parenchyma [17], among other functions. The vulnerability of the BBB to pathogen insults and systemic inflammation might ultimately lead to neural dysfunction, neuroinflammation, and neurodegeneration. Disruption of BBB integrity has been implicated in the pathogenesis of various neurological disorders, including Alzheimer’s disease, Parkinson’s disease, and multiple sclerosis [18].

Despite increasing recognition of the oral–systemic health connection, the specific relationship between AP and BBB integrity remains underexplored, and no direct evidence currently links AP to BBB compromise or neurological disease. However, biological plausibility supported by emerging indirect data [19,20,21,22,23,24] warrants a focused synthesis of the available evidence. Unlike prior reviews addressing periodontitis broadly in relation to systemic and neurological disease, this review focuses specifically on AP and proposes a conceptual framework that organizes the available direct and indirect evidence along the proposed mechanistic sequence linking AP to BBB compromise.

Therefore, this narrative review aims to explore the potential association between AP and BBB integrity, highlighting biological plausibility, existing indirect evidence, and current gaps in knowledge.

## 2. Materials and Methods

An electronic literature search to identify publications related to AP and BBB integrity was conducted using the Scopus and PubMed databases, up to 8 September 2026. The search strategy was as follows:

Search terms were organized into three conceptual blocks and combined using Boolean operators: (1) AP/endodontic infection (“apical periodontitis” OR “periapical lesion” OR “endodontic” OR “periapical abscess” OR “periapical periodontitis”); (2) systemic translocation/inflammation (“systemic translocation” OR “chronic inflammation” OR “mononuclear cell activation”); and (3) BBB/neurological outcomes (“blood–brain barrier” OR “cerebral abscess” OR “neuroinflammation” OR “cerebral inflammation” OR “brain disorders” OR “neurological disorders”). Block 1 was combined with Blocks 2 and 3 using AND, while terms within each block were combined using OR. Additionally, a manual search was performed by screening the reference list of the related papers. Data was extracted, analyzed qualitatively and grouped according to a conceptual framework reflecting the hypothesized sequential steps linking AP to BBB compromise: (i) apical periodontitis; (ii) bacterial and inflammatory mediator translocation from the periapical lesion into the systemic circulation; (iii) peripheral immune activation and elevation of circulating inflammatory markers in AP; and (iv) BBB cellular response and mechanisms of barrier disruption and downstream neurological consequences. This framework guided the organization of the Section 3 into the corresponding thematic sections. Because direct evidence linking AP specifically to BBB compromise remains limited, indirect evidence derived from analogous inflammatory and infectious conditions was incorporated to support biological plausibility at each step, and was explicitly distinguished from direct AP–BBB findings; indirect evidence was further separated into in vitro, animal and clinical studies to reflect differing degrees of translational proximity to the proposed mechanism.

Inclusion criteria: peer-reviewed publications in English reporting on the relationship between AP or AP-associated pathogens and neuroinflammation, BBB integrity, or systemic inflammatory mechanisms relevant to CNS disease, including animal models of induced AP or induced periodontitis involving AP pathogens, in vitro studies using AP-associated bacterial species or patient-derived sera in BBB cellular models, and clinical studies reporting systemic inflammatory markers or CNS outcomes in patients with AP.

Exclusion criteria: publications not available in full text, case reports unrelated to CNS complications of AP, editorials, opinion pieces, and conference abstracts.

Titles and abstracts retrieved from the search were independently screened by two reviewers (CB, MJB) to assess eligibility against the predefined inclusion criteria. Full texts of potentially eligible articles were subsequently retrieved and evaluated in detail. Disagreements regarding study inclusion were resolved by consensus. Given the narrative nature of this review, no formal risk of bias assessment was performed; however, the level of evidence (direct/indirect evidence and human/animal/in vitro studies) of each study type is discussed throughout the manuscript.

To minimize selection bias, eligibility criteria were established a priori and applied consistently across databases, and reference lists of included articles. Findings from in vitro, animal, and clinical studies were narratively synthesized and organized according to the mechanistic pathways proposed to link AP and BBB compromise, distinguishing direct evidence from indirect, disease-analogous evidence where applicable.

For the purposes of this review, direct evidence was defined as studies that specifically investigated apical periodontitis in combination with a direct measure of BBB structural or functional integrity (e.g., permeability assays, transendothelial electrical resistance, or tight-junction protein expression), and that established a causal relationship between AP and BBB compromise. Indirect evidence was defined as studies that (i) reported AP-associated systemic or neuroinflammatory effects without directly assessing BBB integrity (e.g., cytokine or neuroinflammatory markers in brain tissue); (ii) investigated BBB compromise in the context of analogous inflammatory or infectious oral conditions, such as periodontitis, rather than AP itself; or (iii) demonstrated only an association between AP and BBB compromise (i.e., cross-sectional studies). This distinction was applied consistently throughout the Section 3 to differentiate findings that directly implicate AP in BBB dysfunction from evidence supporting biological plausibility through related mechanisms or conditions.

## 3. Discussion

### 3.1. AP Microbiology and Bacterial Translocation

The initial intracanal infection provides a favorable environment for establishing and proliferating a multispecies biofilm, primarily Gram-negative bacteria [25,26]. Next-generation sequencing techniques have confirmed that these infections are characterized by a higher abundance (>5%) of bacterial genera, including *Fusobacterium*, *Pseudoramibacter*, *Porphyromonas*, *Prevotella*, *Lactobacillus*, *Bacteroides*, and *Ralsonia* [27,28,29,30]. *Porphyromonas* spp. (*Porphyromonas endodontalis* and *Porphyromonas gingivalis*) are key pathogens due to their virulence factors, prevalence, and participation in the stability of the bacterial community [3,5,25]. Specifically, *P. endodontalis* has been detected in 56% to 62% of asymptomatic AP cases and in 40% to 69% of AP symptomatic cases [3], and it is recognized as a dominant and metabolically active bacterium in intracanal infections [27].

The presence of *P. endodontalis* DNA in the periapical tissues of teeth with symptomatic and asymptomatic AP [31] marks a significant step in bacterial translocation beyond the root canal system, occurring either as free-floating planktonic cells causing bacteremia or within immune cells [3,4,16]. Bacteremia produced by AP is unlikely a cause of chronic systemic inflammation since most bacterial species are rapidly killed by reactive oxygen species at the surface of erythrocytes and digested by local phagocytes in the liver and the spleen [32]. However, intracellular bacteria can invade and proliferate within the cytoplasm or vacuoles in different types of mammalian cells (e.g., monocytes and endothelial cells) [33]. Within these cells, they primarily conceal themselves within autophagosomes, where they have adapted to evade phagocytic killing. Therefore, intracellular bacteria likely represent a plausible mechanism that facilitates the systemic dissemination of bacteria.

Indirect evidence supports the systemic translocation of AP pathogens and their PAMPs beyond the periapical environment [3,5]. In this regard, it is important to distinguish between three types of evidence with different biological implications: detection of bacterial DNA, detection of viable bacteria in peripheral blood, and evidence of intracellular bacterial translocation. Regarding bacterial DNA detection, a clinical study related to AP has identified DNA from *P. endodontalis* within peripheral mononuclear cells [3], indicating microbial exposure but not confirming the presence of viable organisms, as DNA may persist following bacterial death without retaining infectivity or equivalent proinflammatory capacity. Specifically, total bacterial DNA was detected in 100% of peripheral blood mononuclear cell (PBMC) samples from both AP and healthy individuals, but median bacterial loads were significantly higher in AP subjects than in controls (*p* < 0.05); *P. endodontalis* DNA was identified in 50% of PBMC samples in both groups, with a trend toward higher loads in AP (262.3 vs. 158.8 DNA copies/mL; *p* > 0.05), Furthermore, individuals with endodontic lesions exhibit higher serum levels of anti-*P. endodontalis* IgG antibodies compared to those without such lesions [5]. In that study, intracanal detection frequencies were 33.3% for *P. endodontalis* and 22.9% for *P. gingivalis*; serum anti-*P. endodontalis* IgG was significantly elevated in AP patients versus controls (*p* = 0.02), and intracanal *P. endodontalis* detection was associated with higher endotoxemia within the AP group (*p* = 0.01). Regarding viable bacteria in peripheral blood, a recent study reported that among *Fusobacterium nucleatum*, *P. gingivalis*, and *P. endodontalis*, only the latter was detected in peripheral blood samples from individuals with periodontitis and healthy controls, with significantly higher bacterial loads observed in the periodontitis group, supporting the translocation potential of *P. endodontalis* [34]. Regarding intracellular bacterial translocation, in vitro studies related to periodontitis have shown that *P. gingivalis* (a key pathogen of both periodontitis and AP) can be embedded in vacuoles and internalized in epithelial cells [35]. Similarly, AP-related in vitro studies have shown that *P. endodontalis* induces angiogenesis, activates the pro-inflammatory response in endothelial cells, and can invade endotheliocytes [5,33].

These findings underscore the ability of endopathogens to invade host cells and disseminate systemically, raising the possibility that local oral infections may contribute to the progression of distant, systemic diseases.

### 3.2. Peripheral Inflammation and Immune Activation in AP

Although inflammation is crucial for immune surveillance and host defense, a chronic low-grade inflammatory state is a risk factor for inflammatory non-communicable diseases. Baseline levels of inflammatory markers (C-reactive protein, cell adhesion molecules, TNF-α, or IL-6, among others) are associated with a risk of future myocardial infarction, stroke, and cardiovascular death among apparently healthy populations [4,5]. Additionally, higher circulating levels of proinflammatory interleukins have been related to Alzheimer’s disease and numerous other diseases of the central nervous system (CNS) [36,37].

Clinical AP studies have consistently demonstrated an association between AP and elevated systemic inflammatory markers [4,5,16,38]. Available evidence shows that serum inflammatory marker levels, such as C-reactive protein, IL-6, and matrix metalloproteinases (MMPs), among others, are significantly elevated in patients with AP compared to healthy controls [4]. Furthermore, treatment of periapical lesions can significantly reduce serum inflammatory marker levels in systemically healthy patients [39,40], which constitutes suggestive evidence of a potential causal link between AP and systemic inflammation. It has been hypothesized that this low-grade chronic inflammatory state in AP might be due to inflammatory or bacterial translocation from the oral niches.

Taken together, this body of evidence moves beyond simple cross-sectional association and represents the strongest available support for a biologically plausible link between AP and systemic inflammatory burden, although randomized controlled trials controlling for confounding factors are still needed to establish definitive causality.

Consistent with this translocation hypothesis, oral bacteria and their associated PAMPs may drive the activation of circulating immune cells. Both Gram-negative intracellular bacteria and PAMPs, like LPS, have been associated with mononuclear cell activation [41,42] (Figure 1). In AP, mononuclear peripheral cells, as well as isolated monocytes, display a proinflammatory profile with higher secretory levels of TNF-α, IL-1β, and IL-6 [43,44]. This cell activation may be partially explained by the recognition of PAMPs through pattern recognition receptors, such as Toll-like receptors (TLRs) [43]. When these receptors recognize PAMPs, macrophages are activated to the M1 phenotype [41], which produces proinflammatory cytokines and chemokines, enhancing the immune response [4,5,16,38]. While the mechanisms above relate primarily to Gram-negative PAMPs such as LPS, an analogous macrophage-activating pathway has recently been described for Gram-positive bacteria, mediated by mitochondrial dysfunction. In a lipoteichoic acid (LTA)-induced macrophage model simulating the AP inflammatory environment, LTA from Enterococcus faecalis, a pathogen strongly associated with refractory AP, upregulated inflammatory mediators (NLRC4, iNOS, NF-κB, Caspase-1) alongside mitochondrial dysfunction, characterized by morphological disruption, dysregulated dynamics, impaired autophagy, and elevated ROS [45]. These findings were corroborated in the same cellular model using pharmacological inhibition of mitochondrial fission with Mdivi-1. Mdivi-1 restored mitochondrial function and attenuated the inflammatory response against LTA stimulation, suggesting mitochondrial dynamics as a targetable mechanism in refractory AP driven by Gram-positive pathogens [45]. Given the translocation of oral bacteria and PAMPs into the circulation, this Gram-positive-driven mechanism may extend to circulating monocytes exposed to LTA released from periapical E. faecalis. While mitochondrial function has not yet been examined in peripheral monocytes from AP patients, this cell population has been shown to upregulate TLR-2 [46], the principal receptor for LTA recognition, supporting the plausibility of this pathway operating systemically.

Due to the persistence of endodontic infection without proper treatment, the chronicity of a low-grade inflammatory state might lead to dysregulation of the immune system, altered levels of circulating inflammatory biomolecules, and facilitate the progression of non-communicable diseases. Substantial evidence has established an association between AP and systemic diseases such as diabetes mellitus, osteoporosis, rheumatoid arthritis, autoimmune hepatitis and nephritis, cardiovascular diseases, gastrointestinal diseases, and adverse pregnancy outcomes [47,48], and it might potentially contribute to neuroinflammation and blood–brain barrier permeabilization.

### 3.3. Could Endodontic Pathogens Activate the Inflammatory Pathways in the BBB?

Structurally, the BBB comprises brain endothelial cells with continuous intercellular tight junctions, pericytes, astrocyte end-feet, and the capillary basement membrane [17] (Figure 2). Beyond its role in immune surveillance by limiting immune cell access to the brain [17], each of these cellular components can independently respond to circulating inflammatory insults, contributing to barrier disruption through distinct mechanisms, as detailed below.

Brain endothelial cells express receptors that enable them to respond to systemic inflammation (IL-1β, IL-6, and TNF-α, among others) [49,50] (Figure 2). They express TLR2, TLR4, and TLR6 for Gram-positive LTA and Gram-negative LPS [49]. When BBB endothelial cells detect inflammatory products, such as those induced by AP pathogens, they react by upregulating pro-inflammatory cytokines (IL-6, IL-8), endothelial cell adhesion molecules (ICAM-1, sVCAM-1), immune cell recruitment proteins (MCP-1, CCL2, CCL5, CXCL10), angiogenic factors (VEGF), and growth factors inducing immune cell proliferation (G-CSF, GM-CSF) [50]. Additionally, LPS induces endothelial cell necroptosis mediated by TNF-α [51]. Under inflammatory conditions, brain endothelial cells also release metalloproteinases (MMP-3, MMP-9, and MMP-12) [52], which are associated with BBB disruption through proteolytic activity (Figure 2). Similarly, pericytes of the BBB respond to inflammatory stimuli (IL-1β and/or TNF-α) by secreting IL-6, IL-8, ICAM-1, VCAM-1, CCL5, and CX3CL1 [53], as well as MMP-9 [54,55] (Figure 2). Finally, astrocytes respond to systemic inflammatory stimuli by activation, enhanced number of cellular processes, and proliferation (known as astrogliosis), followed by astrocyte loss [56,57], and structural changes in astrocytic end-feet [58] (Figure 2). Systemic inflammatory stimuli also induce pro-inflammatory and cytotoxic pathways in astrocytes [59], and astrocytes can produce a range of cytokines, including IL-1β, IL-6, TNF-α, and prostaglandins [60]. These pro-inflammatory molecules and reactive species activate a series of signaling pathways that can damage tissues and activate cell death pathways. Compromised BBB integrity implies increased barrier permeability and disruption of tight junctions in the endothelial cell layer [51,53].

The consequences of a compromised BBB include neural dysfunction, neuroinflammation, and neurodegeneration. According to the WHO, 1 in 3 people are affected by neurological conditions, which are currently the leading cause of illness and disability worldwide, with the overall burden of disability, disease, and premature death caused by these conditions increasing by 18% over the last 35 years (WHO, 2024). Beyond the neurodegenerative disorders noted above, BBB breakdown is also a hallmark of Huntington’s disease and amyotrophic lateral sclerosis [18], as well as non-neurodegenerative CNS conditions such as depression and anxiety disorders [61]. Although in many of these diseases it remains unclear whether BBB disruption is a causal factor, its degradation is at least a contributing factor to disease progression and exacerbation, as changes in BBB integrity and neuropathology drive each other in a self-perpetuating manner [62].

### 3.4. Indirect Evidence of AP and BBB Compromise: Animal Studies

Accumulating preclinical evidence, derived from both periodontal pathogen animal models and AP-specific animal models, suggests that oral infection and associated pathogens may play a role in sustained neuroimmune activation and BBB compromise; however, direct evidence specifically linking AP to BBB disruption remains scarce. Indirect evidence from periodontal pathogen animal models have demonstrated that *P. gingivalis* trespasses the BBB [22]. Moreover, the oral inoculation of *P. gingivalis* significantly increases microglia and astrocyte activation, expression of proinflammatory cytokines such as interleukin-1β (IL-1β) and IL-6, and causes neuronal damage in a mouse model [20]. In addition, mice infected orally with *P. gingivalis* exhibit brain colonization by the pathogen and show classic features of Alzheimer’s disease, including neuroinflammation, neurodegeneration, tau phosphorylation, and intracellular and extracellular amyloid-β plaques and neuronal fibrillary tangles [21,24]. It should be noted that these findings derive from animal periodontitis models and their extrapolation to AP requires caution, as the two conditions differ in their microbial ecology, anatomical location, and inflammatory dynamics.

Given the high genetic similarity resulting from their close phylogenetic relationship, it may be hypothesized that *P. endodontalis* could exhibit characteristics similar to *P. gingivalis* in terms of its potential to induce endothelial damage and increase BBB permeability. Regarding AP-specific animal models, in a pioneering study, researchers using a rodent model of induced AP in pregnant Sprague–Dawley rats found significantly elevated levels of the proinflammatory cytokines IL-6, IL-1β, and TNF-α in the offspring, alongside reduced levels of myelin basic protein (MBP) and norepinephrine in the hippocampus and prefrontal cortex [19]. These findings suggest that inflammatory insults originating from localized oral infections during gestation may affect fetal neurodevelopment by altering neurochemical and myelination processes. Similarly, adult Wistar rats with AP exhibited elevated concentrations of TNF-α, IL-1β, and IL-6 within critical brain regions such as the hippocampus and frontal cortex [23]. These proinflammatory mediators are believed to disrupt BBB integrity or act through peripheral immune signaling, ultimately leading to the activation of microglia and astrocytes, the cellular drivers of chronic neuroinflammation and brain impairment [63,64]. Finally, the induction of experimental AP in rodent models demonstrated that *Streptococcus mutans*, particularly the Cnm-positive strain, can migrate from infected apical lesions to the bloodstream and exacerbate cerebral hemorrhage in stroke-prone spontaneously hypertensive rats [65]. Collectively, while these AP-specific animal studies provide valuable mechanistic insights into the neuroinflammatory consequences of apical infection, none has directly assessed BBB integrity as a primary outcome, highlighting a critical gap in the current evidence base that warrants further investigation.

### 3.5. Indirect Evidence of AP and BBB Compromise: Clinical Studies

Bergandi et al. [66] reported the first study to demonstrate AP-associated mechanisms within the context of BBB inflammatory biology. At the clinical level, patients with AP exhibited significantly elevated serum levels of ZO-1 and Claudin-5 (tight junction proteins indicative of endothelial barrier damage) compared to healthy controls, with levels progressively decreasing following successful root canal treatment, suggesting that systemic inflammation in AP is reflected not only in circulating cytokines but also in proteins associated with endothelial barrier disruption. Extending these findings to an in vitro model, the same study demonstrated that exposure of hCMEC/D3 human brain microvascular endothelial cells to sera from untreated AP patients significantly increased endothelial permeability to dextran-FITC and decreased transendothelial electrical resistance (TEER), indicating BBB dysfunction, while sera collected following successful endodontic treatment restored barrier integrity to control levels and immunofluorescence analysis confirmed recovery of ZO-1 protein expression. Although these findings are preliminary and derive from a simplified cellular model that does not fully recapitulate the complexity of the BBB in vivo, they constitute the first experimental evidence that the systemic inflammatory milieu associated with AP can compromise BBB function in vitro, and that endodontic treatment may reverse this effect.

While infrequent, AP, particularly when undiagnosed or inadequately treated, has been implicated in severe acute CNS infections, such as brain abscesses, subdural empyema, meningitis, and cavernous sinus thrombosis [67,68,69]. The posterior maxillary teeth, especially molars and premolars, are frequently involved in these cases due to their anatomical adjacency to vascular plexuses and sinus cavities, which can facilitate microbial transit into intracranial spaces [67,68,69]. Microbiological analysis of intracranial specimens, including abscess and autopsy tissues, has revealed a predominance of anaerobic and facultative intraoral pathogens. Frequently isolated organisms include *Fusobacterium nucleatum*, *Streptococcus intermedius*, *Prevotella* spp., and *Actinomyces* spp., all typical members of the endodontic microbiota [70,71,72].

Consistent with these case reports, a recent systematic review by da Conceição Francisquini et al. [73] compiled 12 studies (case reports, case series, animal experiments, and one cohort study) linking AP with neuroinflammation and brain disorders, with some cases resulting in death. This synthesis reinforces the biological plausibility of pathways connecting periapical infection to the CNS and supports AP as a potential peripheral trigger of neuroinflammatory pathology.

These findings emphasize the importance of timely recognition and proper management of AP in acute cases presenting with clinical symptoms and radiographic evidence of periapical lesions. Despite their rarity, these complications (acute CNS infections) carry a high risk of morbidity and mortality [68,69,70] and underscore the relevance of interdisciplinary awareness in both dental and medical settings.

## 4. Conclusions

Although biological plausibility is well supported and preliminary in vitro and animal evidence has begun to emerge, a direct causal relationship between AP and BBB dysfunction has not yet been established. Accordingly, in vivo preclinical studies and prospective clinical studies are still needed to establish a clear causal relationship between AP and BBB compromise. Given the high prevalence and significant global burden of neurological disorders, investigating the influence of apical periodontitis on blood–brain barrier integrity represents a promising avenue for future research.

## 5. Future Directions

While the previous sections have outlined a plausible biological pathway linking AP to BBB compromise, direct evidence in humans or animals remains absent, representing a critical gap in the literature. Establishing a clear cause–effect relationship between these two conditions is inherently challenging for several interconnected reasons. First, the BBB is subject to multifactorial regulation, and isolating AP as an independent contributing factor requires meticulous control over confounding variables, including other sources of systemic inflammation, genetic predispositions, and co-morbidities, all of which can independently influence barrier integrity. Second, demonstrating temporality, a fundamental requirement for causal inference, necessitates longitudinal study designs capable of capturing the temporal sequence between AP onset or resolution and subsequent changes in BBB function, which in turn demands considerable time, resources, and participant follow-up. Addressing these challenges will require a convergent and methodologically rigorous research methodology. Future preclinical studies should prioritize AP-specific models that allow direct assessment of the effects of AP-associated inflammation on BBB integrity. Concurrently, further studies should adopt standardized AP diagnostic criteria, quantitative assessment of periapical inflammatory burden, appropriate control groups, rigorous adjustment for confounding variables, and validated markers or functional measures of BBB integrity. The establishment of definitive causal understanding in this field will require the systematic integration of evidence derived from physiologically relevant experimental platforms and rigorously designed clinical investigations.

## Figures and Tables

**Figure 1:**
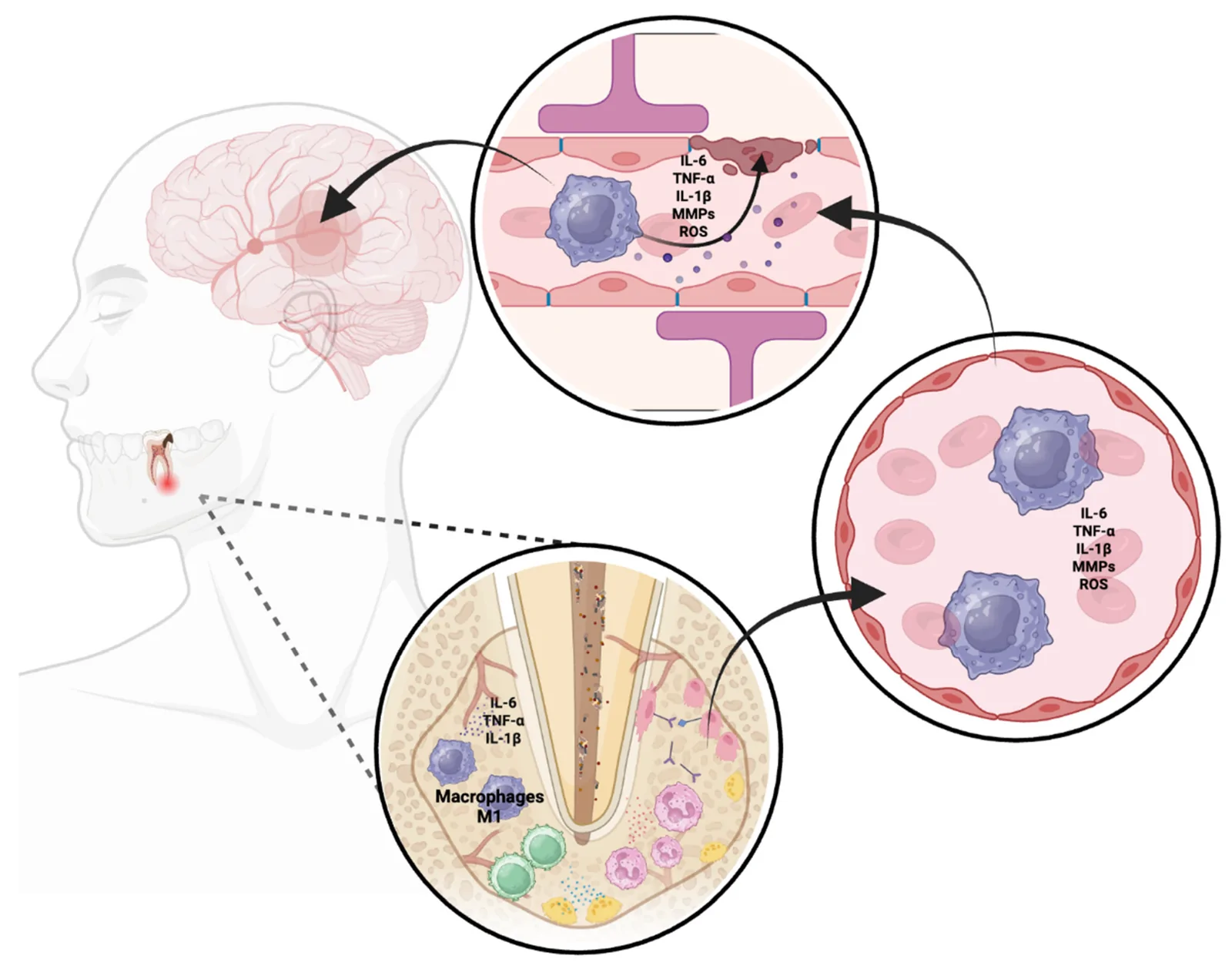
Proposed potential link between apical periodontitis and blood–brain barrier (BBB) compromise. Previous studies have shown an association between apical periodontitis and pro-inflammatory macrophage phenotype, increasing circulating cytokines (e.g., IL-6, TNF-α, IL-1β), matrix metalloproteinases, ROS, and bacterial byproducts. These factors may reach the BBB via the bloodstream, where they can activate endothelial receptors, triggering inflammation, necroptosis, proteolysis, and structural changes. Chronic exposure to these insults may compromise BBB integrity and function. Created in BioRender. Hernández, M. (2026). https://biorender.com/od4znyr.

**Figure 2:**
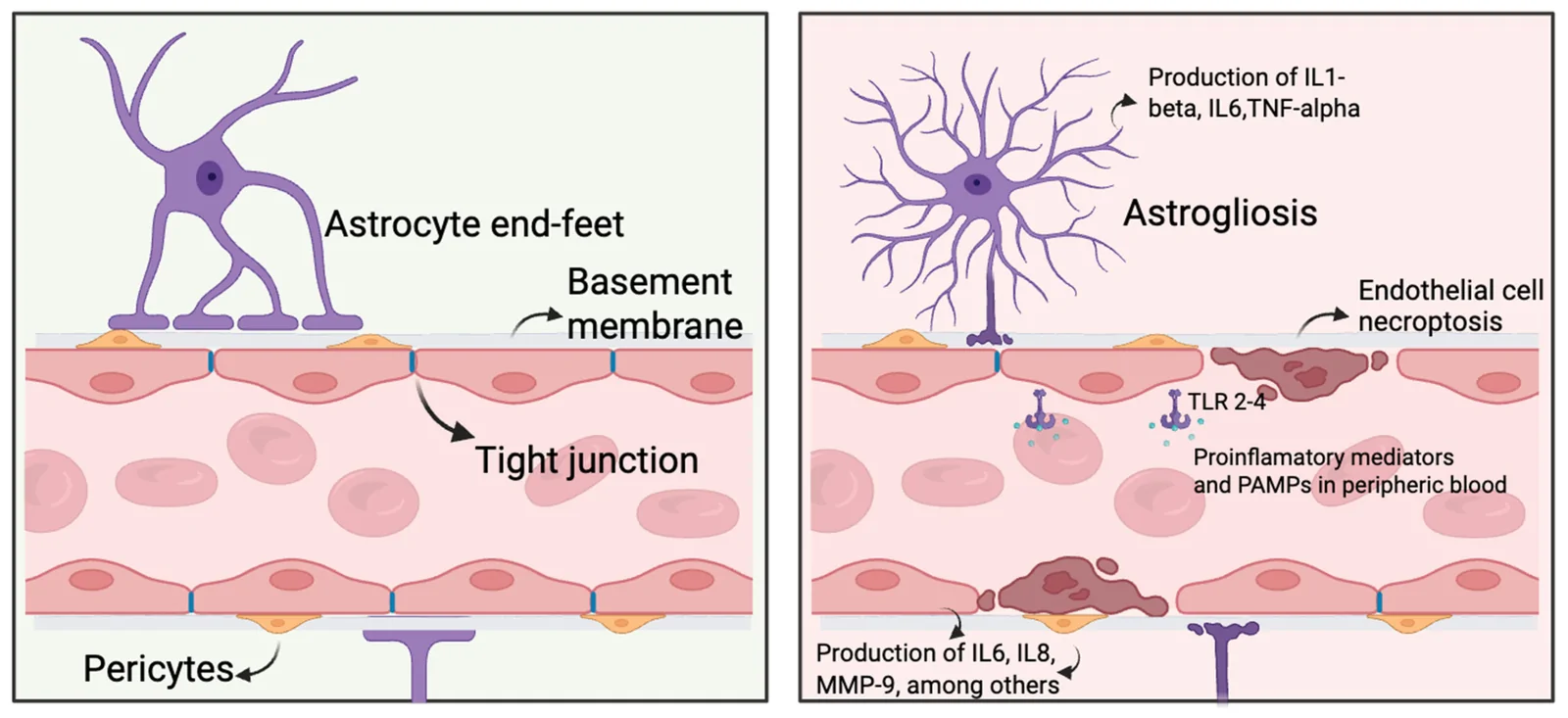
Proposed mechanism by which systemic inflammation compromises blood–brain barrier (BBB) integrity, based on evidence synthesized from the reviewed literature. The schematic contrasts a healthy BBB, composed of brain endothelial cells connected by continuous tight junctions, pericytes, astrocyte end-feet, and a capillary basement membrane, with a BBB affected by systemic inflammation. According to this proposed mechanism, activation of toll-like receptors by systemic inflammation induced by AP and their pathogen-associated molecular patterns (PAMPs) triggers a complex response in the BBB, including the activation of proinflammatory pathways, endothelial cell necroptosis, and astrogliosis, which compromise BBB integrity. Created in BioRender. Hernández, M. (2026). https://biorender.com/k685y4m.

## Data Availability

No new data were created or analyzed in this study. Data sharing is not applicable to this article.

## Abbreviations

AP	Apical periodontitis
BBB	Blood–brain barrier
CCL2	Chemokine (C-C motif) ligand 2
CCL5	Chemokine (C-C motif) ligand 5
Cnm	Collagen-binding protein Cnm (Streptococcus mutans)
CNS	Central nervous system
CX3CL1	Chemokine (C-X3-C motif) ligand 1
CXCL10	Chemokine (C-X-C motif) ligand 10
FITC	Fluorescein isothiocyanate
G-CSF	Granulocyte colony-stimulating factor
GM-CSF	Granulocyte–macrophage colony-stimulating factor
ICAM-1	Intercellular adhesion molecule 1
IgG	Immunoglobulin G
IL-1β	Interleukin-1 beta
IL-6	Interleukin-6
IL-8	Interleukin-8
iNOS	Inducible nitric oxide synthase
LPS	Lipopolysaccharide
LTA	Lipoteichoic acid
MBP	Myelin basic protein
MCP-1	Monocyte chemoattractant protein-1
MMP	Matrix metalloproteinase (MMP-3, MMP-9, MMP-12)
NF-κB	Nuclear factor kappa B
NLRC4	NLR family CARD domain-containing protein 4
PAMPs	Pathogen-associated molecular patterns
PBMC(s)	Peripheral blood mononuclear cell(s)
ROS	Reactive oxygen species
sVCAM-1	Soluble vascular cell adhesion molecule 1
TEER	Transendothelial electrical resistance
TLR	Toll-like receptor (TLR2, TLR4, TLR6)
TNF-α	Tumor necrosis factor-alpha
VEGF	Vascular endothelial growth factor
WHO	World Health Organization
ZO-1	Zonula occludens-1
